# The long journey to a Systems Biology of neuronal function

**DOI:** 10.1186/1752-0509-1-28

**Published:** 2007-06-13

**Authors:** Nicolas Le Novère

**Affiliations:** 1 EMBL-EBI, Wellcome-Trust Genome Campus, CB10 1SD Hinxton, UK

## Abstract

Computational neurobiology was born over half a century ago, and has since been consistently at the forefront of modelling in biology. The recent progress of computing power and distributed computing allows the building of models spanning several scales, from the synapse to the brain. Initially focused on electrical processes, the simulation of neuronal function now encompasses signalling pathways and ion diffusion. The flow of quantitative data generated by the "omics" approaches, alongside the progress of live imaging, allows the development of models that will also include gene regulatory networks, protein movements and cellular remodelling. A systems biology of brain functions and disorders can now be envisioned. As it did for the last half century, neuroscience can drive forward the field of systems biology.

## 1 Modelling nervous function, an ancient quest

Neurosciences have a long and successful tradition of quantitative modelling, where theory and experiment have always formed a happy couple. The work of Warren Sturgis McCulloch and Walter Pitts on formal neural networks [1] gave rise to one of the best examples of cross-fertilising scientific fields, which resulted in many advances both in information technology and cognitive science. Almost as soon as digital computers became available, they were used by neuroscientists to quantitatively test their theories. One of the first numerical simulations in biology was the famous model of Alan Lloyd Hodgkin and Andrew Huxley [2], that explained the propagation of action potentials along axons – and as a by-product postulated the existence of ion channels in the membrane, before the experimental proof of their existence. Quantitatively describing a cellular behaviour emerging from the interaction between two different molecular components, a potassium and a sodium channels, the model of Hodgkin-Huxley can arguably be seen as the beginning of computational systems biology [3].

To accurately model neuronal function presents many challenges, and stretches the techniques and resources of computational biology to their limits. The molecular and cellular events mediating neuronal transmission span several spatial and temporal scales. While the signal received from a glutamatergic terminal is decoded by a 500 nanometer wide dendritic spine [4], the resulting action potential can be propagated along axons up to 1 metre long. Understanding synaptic function also means deciphering the effect of conformational transitions of ion channels, taking place on the microsecond range, onto long-term synaptic modifications lasting several weeks. Moreover, most assumptions used to simplify modelling in other fields of cell biology, such as homogenous concentrations and spatial isotropy are inappropriate. The geometry of subcellular compartments strongly affects their functions [5], as does the relative location of molecular partners and their diffusion. Finally, the morphology of neurons changes over time, and itself depends on the activity of the neurons [6,7]

## 2 Travelling from the ion channel to the brain

With the cable approximation, Wielfrid Rall opened the way to realistic multi-compartment electrical models [8]. This approach assimilates a portion of dendrite to a simple electrical circuit that can then be assembled serially. These models quickly spanned several scales, encompassing synaptic contacts between neurons [9], models of multicellular structures [10], and even of several coupled brain structures [11]. The availability of powerful and easy to use simulators to develop such multi-compartment models, like NEURON [12] and GENESIS [13] allowed the construction of extremely detailed models of neurons. Those models include not only electrical behaviour, but also ion diffusion [14]. Advanced computing facilities now permit the development of large heterogeneous neuronal assemblies, where each neuron possess a realistic geometry and specific electrophysiological properties determined by a given set of ion channels. The most ambitious project in this domain may be the Blue Brain Project [15], which aims to simulate a whole mammalian cerebral cortex using a super-computer. As a proof-of-concept, simulations of a neocortical column containing 10,000 neurons have been run. In parallel to the development of electrical models, neurobiologists started to model neuronal signalling using the concepts of chemical kinetics, already widely used in biochemistry [16]. The coupling of reaction kinetics with single particle diffusion and realistic spatial representation now allows the simulation of neuronal signalling at a level of detail only dreamt of before [17]. At the end of last century, two decades of molecular and cellular neurobiology had demonstrated that to reach a comprehensive understanding of neuronal signalling, we ought to consider both electrical and biochemical signal transduction [18,19].

## 3 What are the roadblocks?

Although computational systems neurobiology is still far ahead of other fields when it comes to multi-scale, multi-algorithms modelling, the coupling of signalling pathways, electrical dynamics and ionic diffusion is still infrequent [20]. Even more serious is the fact that some crucial cellular functions or behaviours are barely considered at all when it come to quantitative modelling. Modifications of gene expression [21] and protein translation [22] have been largely studied in synaptic function and plasticity, or in the symptomatology of neuronal diseases [23]. Due to the different time-scales involved, and the difficulty of building hybrid models able to provide continuous descriptions of electrical, metabolic and signalling events together with stochastic or even logical descriptions of gene regulatory networks [24], those aspects of neuronal physiology are mainly considered separately. Cell remodelling has also been generally ignored, whether at the level of the synapse, the spine or the neuronal process, despite an abundant literature showing its importance in neuronal function.

The recent availability of new types of quantitative data should help to expand the models in new directions. On the large-scale front, functional genomics approaches such as microarrays [25] or proteomics [26], but also systematic application of more classical approaches such as *in situ *hybridization [27] should make the models more accurately reflect brain function and dysfunction. Other cutting-edge technologies like single-particle tracking in living cells [28] will allow the development of more realistic models, and will enable the investigation of the role of micro-domains and supra-macromolecular complexes.

## 4 Let's hit the road

With the general improvement of physical health in developed countries, the relative importance of neuropathology is growing. Mental illnesses are becoming significant public health concerns, schizophrenia, for example, having an incidence approaching 0.5–1% of the population [29]. The general ageing of the population also increases the incidence of neurodegenerative disorders such as Parkinson's disease, touching more than 1% after the age of 65 in some countries [30]. Finally, drug addiction, and the associated direct or indirect mortality, remains the most widespread mental disorder and a major worldwide societal problem.

Neurobiology has led the way in computational modelling for over half a century. It is now time to scale up and develop a real systems biology of the nervous system and the associated diseases. The quantitative information is either already available or on its way. Although progress has to be made on the multi-scale and model integration fronts, the methodology is essentially here. The computing power required is matched by the latest generation of super-computers. Neurobiologists have no excuse not to be at the forefront of computational systems biology.
